# Supplementary material for: Identification and Validation of Novel Potential Pathogenesis and Biomarkers to Predict the Neurological Outcome after Cardiac Arrest
Source: Brain Sci. 2022 Jul 15;12(7):928. doi: 10.3390/brainsci12070928 (PMC9316619; doi:10.3390/brainsci12070928)
Supplement: Supplementary file 1 [file brainsci-12-00928-s001.zip › brainsci-1766493-supplementary.pdf]

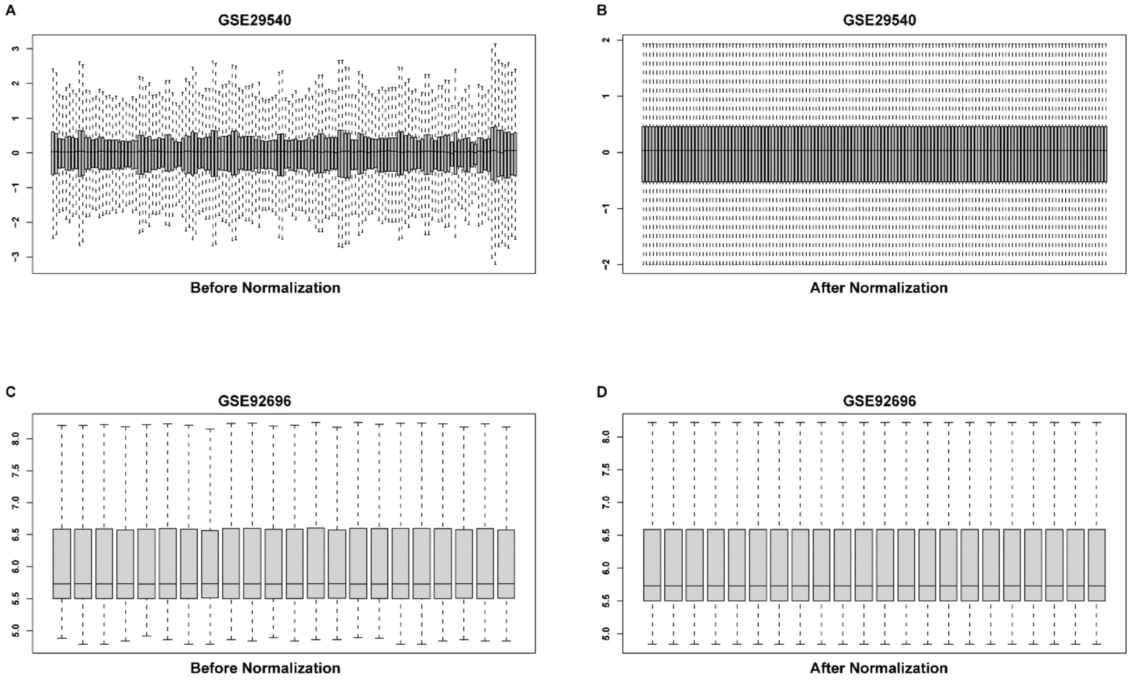

Figure S1. Normalization of datasets. A. The expression distribution box diagram of each sample in GSE29540 before normalization. B. The expression distribution box diagram of each sample in GSE29540 after normalization. C. The expression distribution box diagram of each sample in GSE92696 before normalization. D. The expression distribution box diagram of each sample in GSE92696 after normalization.

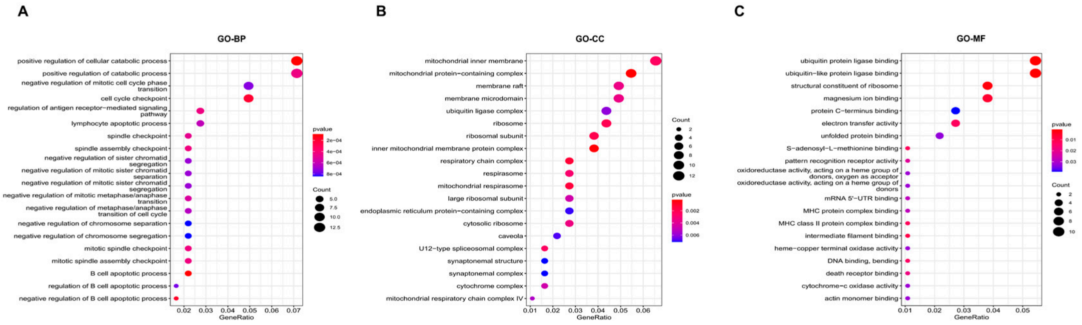

Figure S2. Enrichment analysis of DEGs. A-C. GO Enrichment analysis of overlapping DEGs, including BP (A), CC (B), and MF (C). The top20 terms were displayed. The top20 terms were displayed.

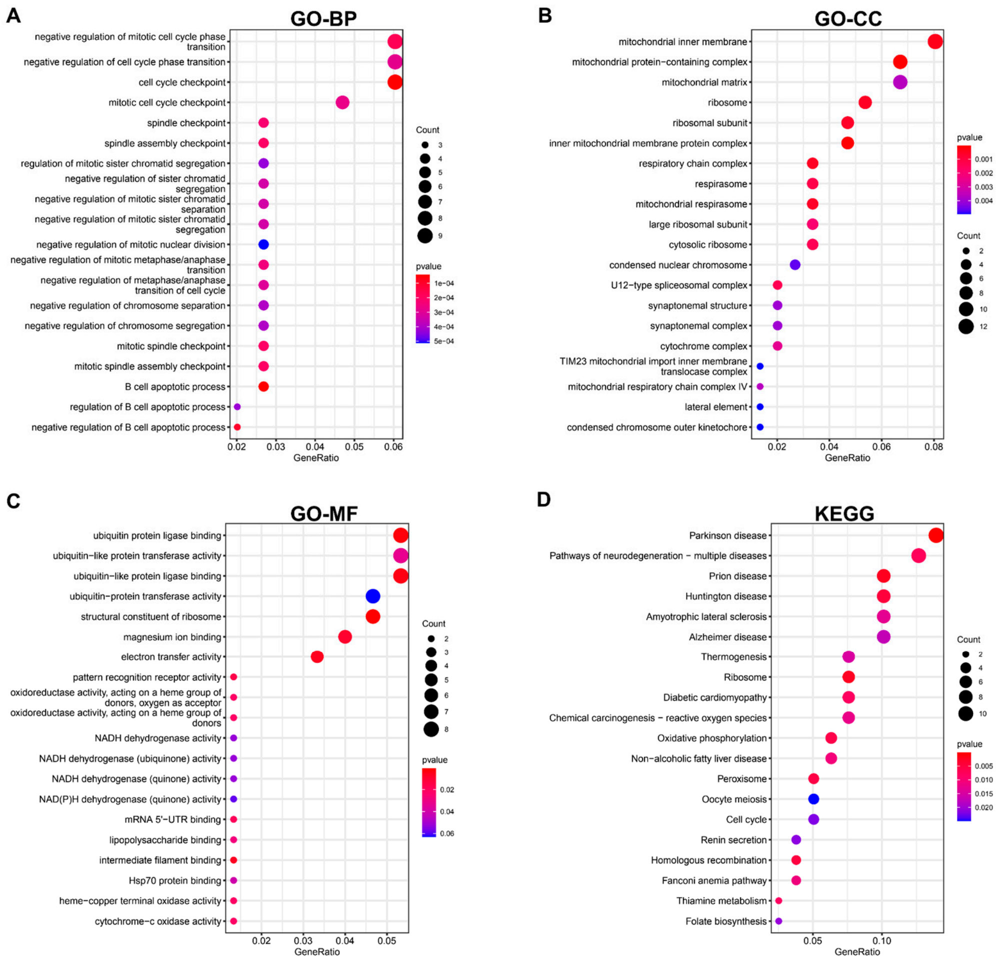

Figure S3. Enrichment analysis of DEGs from key modules. A-C. GO Enrichment analysis of DEGs, including BP (A), CC (B), and MF (C). D. KEGG analysis of DEGs.

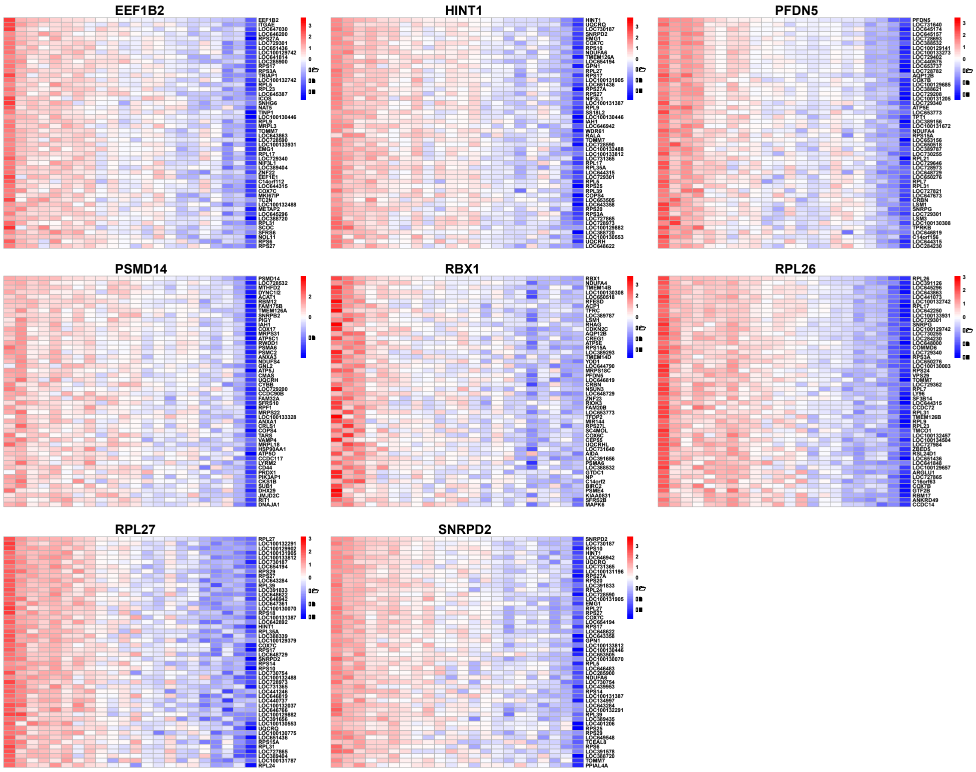

Figure S4. The Co-expression analysis of hub genes.

The top50 genes most positively associated with the indicated hub genes were shown separately in the heatmap.
